# Factors Affecting the Outcome of Vitrectomy With Internal Limiting Membrane Peeling for Myopic Foveoschisis

**DOI:** 10.1155/joph/2774963

**Published:** 2025-02-13

**Authors:** Lie Yang, Jialin Wang, Lu Zhao, Zhuohua Zhou, Yingxiang Huang, Yanling Wang

**Affiliations:** Department of Ophthalmology, Beijing Friendship Hospital, Capital Medical University, Beijing, China

## Abstract

**Purpose:** To evaluate the short- and long-term effect of pars plana vitrectomy (PPV) with internal limiting membrane (ILM) peeling on visual acuity and macular morphology in myopic foveoschisis (MF) patients and to identify potential prognostic factors.

**Methods:** We retrospectively analyzed the clinical data of patients with MF who underwent PPV with ILM peeling by the same senior fundus surgeon at Beijing Friendship Hospital from January 2016 to December 2022. The peeling was strategically centered on the macular fovea, extending to encompass the superotemporal and inferotemporal vascular arcades. Univariate analysis and multivariate logistic regression analysis were conducted to screen out the prognostic factors.

**Results:** 36 eyes of 36 consecutive patients were analyzed in total. Best-corrected visual acuity (BCVA) improved significantly from 1.10 ± 0.61 logMAR to 0.78 ± 0.58 logMAR (*p*=0.031). 23 eyes (63.89%) had postoperative BCVA improved ≥ 2 Snellen lines. The mean central fovea thickness (CFT) decreased from 427.14 ± 255.91 μm to 155.85 ± 67.33 μm (*p* < 0.001). 18 and 16 eyes achieved partial and complete retinal reattachment, respectively, as follows. The twelfth month postoperatively was a threshold to influence the resolution of MF significantly, but it did not affect the visual outcome. Multiple logistic regression showed CFT (OR = 1.007, 95% CI = 1.001, 1.013, *p* value = 0.034) remained significant to predict the complete retina resolution. For visual acuity, integrated ellipsoid zone (EZ) band (OR = 0.239, 95% CI = 0.073, 0.783, *p* value = 0.018) might be a significant predictive factor. Subgroup analysis further indicated that in eyes with an intact EZ band, a poorer baseline BCVA was associated with an increase in postoperative BCVA (*p*=0.015). Conversely, in those with disrupted EZ band, all included factors showed no significant difference.

**Conclusion:** The study observed trends in the recovery pattern of the retina following surgery and suggested potential factors that may be associated with improvements in both visual acuity and retinal reattachment. The findings may offer some guidance to ophthalmic surgeons in considering the timing of surgery, although further research is needed to confirm these trends as definitive predictors.

## 1. Introduction

Myopic foveoschisis (MF) is characterized by the splitting and separation of the neurosensory retinal layers in the macula in highly myopic eyes. MF is one of the main complications of pathological myopia. In two population-based studies with large sample sizes, the prevalence of MF among highly myopic eyes was 32.9%, accounting to the most common macular change [1, 2]. Pathologically, the splitting and separation of the retinal layers of MF is due to anteroposterior vitreous traction and rigidity of the internal limiting membrane (ILM). As progression, disrupted ellipsoid zone (EZ), lamellar or full-thickness macular hole (FTMH), and retinal detachment may occur secondary to MF. Although a few cases have reported spontaneous improvement of MF [3, 4], a more recent population-based study shows that eyes with macular retinoschisis tend to progress more extensively in the following time [5].

The treatment for MF is primarily surgery, including macular buckling, posterior scleral reinforcement, pars plana vitrectomy (PPV), or the combination. Macular buckle (MB) surgery is one of the pivotal techniques in the treatment of myopic traction maculopathy (MTM), and it has been established that MB also offers good safety and postoperative outcomes in eyes with MTM, despite the technical challenges associated with the procedure, such as incomplete resolution or recurrence of MTM, choroidal detachment, subretinal hemorrhage, or postoperative buckle compression. Historically, retinal pigment epithelium atrophy was considered the primary postoperative complication of MB; however, a recent long-term follow-up study has found that MB surgery does not alter the progression of atrophic or pigmentary changes in high myopia maculopathy [6]. PPV with ILM peeling with or without tamponade has been shown an effective treatment for MF, aiming to relieve the vitreoretinal traction on the macula [7–9]. Some scholars posit that when MF progresses to foveal retinal detachment, vitrectomy can prevent the occurrence of macular hole (MH) and hole-related retinal detachments. Additionally, vitrectomy with ILM peeling has demonstrated favorable therapeutic effects and postoperative visual acuity (VA) enhancement in treating various types of MTM, while also preventing the recurrence of tractional retinal detachments. Nevertheless, there remains debate regarding the optimal timing for surgery and the applicability and limitations of different surgical approaches.

Previous studies have identified potential prognostic factors of vitrectomy for MF, including central fovea thickness (CFT) [10], preoperative VA, axial length (AL) [11–13], photoreceptor layer integrity, or choroidal surface regularity [14]. However, most research focused on the linear association between preoperative factors and postoperative VA. Few have explored the prognostic factors of a significant improvement of VA and anatomical outcome after surgery. When the patient should receive surgery and whether he/she would benefit greatly remain key questions in clinical practice.

The present study aimed to identifying the predictive factors for both vision improvement and retinal reattachment for individual patients before vitrectomy. Further, we identified a proper perspective timepoint to observe the effect of vitrectomy treating MF.

## 2. Materials and Methods

### 2.1. Patients and Project Design

This retrospective case series study recruited patients who underwent PPV with ILM peeling for MF from January 2016 to December 2022 at the Department of Ophthalmology of Beijing Friendship Hospital, Capital Medical University (Beijing, China), by the same senior surgeon. The physical examination and optical coherence tomography (OCT) data were regularly examined and recorded before and 1, 3, and 6 months or more after surgery. The study was performed following the tenets of the Declaration of Helsinki as revised in 1989.

The study was focused on middle-aged or older adults (defined as 50 years or older) with severe MF (defined as foveoschisis grade S4 according to Shimada's research [15]) or with FTMH. High myopia was defined as spherical equivalent greater than −6.00 diopters (D) or an AL more than 26.0 mm. All subjects were identified as myopia macular foveoschisis from the appropriate International Classification of Diseases codes. Exclusion criteria included MF due to trauma or other causing reasons rather than high myopia, concomitant ocular diseases such as inflammation, corneal disorders, glaucoma, tumor, moderate or severe cataracts, history of intraocular surgeries except for cataract phacoemulsification with intraocular lens (IOL) implantation, or other retinal disorders including age-related macular degeneration, retinal vascular diseases, and retinal detachment.

Patient demographics, complete ophthalmic examination including best-corrected visual acuity (BCVA), noncontact tonometry, slit-lamp examination, AL measurement by optical biometry using an IOL Master (Carl Zeiss Meditech, Jena, Germany), and assessment of visual significance of lens status were recorded preoperatively and postoperatively. Multimodal imaging including color fundus photography and OCT (Heidelberg Engineering, GmbH, Heidelberg, Germany) data were recorded. A 6 × 6 mm area of the macular region centered on the fovea was examined using spectral domain OCT, including a linear mode and a radial mode, performed using the Spectralis (Heidelberg Engineering, Heidelberg, Germany) platforms. All OCT scans were retrospectively assessed by two trained graders (J.L.W. and L.Y.). The measurements were performed manually using the imaging software (Heidelberg Engineering, Heidelberg, Germany; Software version 5.3) after the tomographs had been reviewed. OCT variables that were recorded included the existence and location of posterior scleral staphyloma, location and height of retinoschisis, thickness of retinoschisis, integrity of the EZ and choroidal surfaces, linear diopter of MH, and the area and height of foveal detachment (FD). Characteristic variables including atrophy, traction, and neovascularization were graded by the new ATN classification system as described by Ruiz-Medrano et al. [16]. OCT identified FD height as the largest distance measured manually between the inner border of the retinal pigment epithelium and the outer border of the neural retina at the foveal area, the FD length as the average distance of detachment area from side to side, and the perpendicular fovea thickness as the retinal thickness at the central fovea. The presence of macular retinoschisis was confirmed and further classified into five stages based on location and size as described before [15]. The presence of posterior staphylomas was identified from color fundus photographs, OCT, or B-scan ultrasonographic images by visualizing the border of the ectasia and irregular protrusion or conical elongation of the globe [17, 18]. The location and types of the staphylomas were determined based on the classification reported by Ohno-Matsui [19].

All patients underwent surgery executed by a single, highly experienced surgeon (Y.L.W.) with over a decade of expertise in PPV. The procedure commenced with a standard three-port PPV, utilizing 25-gauge instrumentation. Following the triamcinolone-assisted core vitrectomy to discern the posterior hyaloid, a comprehensive vitrectomy ensued, culminating in posterior vitreous detachment. The ILM was meticulously stained with indocyanine green, enhancing its visualization and allowing for precise dissection with ILM forceps. The peeling was initiated at the superotemporal and inferotemporal vascular arcades and carefully conducted centripetally toward the macular fovea, ensuring a meticulous and controlled approach to address the full extent of the ILM. Concluding the intervention, a carefully selected tamponade was judiciously injected to facilitate and support the reattachment of the retina. Postoperatively, patients who received gas and silicone oil tamponade were instructed to maintain a face-down position, with the duration determined based on individual clinical considerations.

The existence of concomitant MH (lamellar or full), epiretinal membrane (ERM), vitreomacular traction (VMT), or FD was also recorded. Operative variables included the choice of tamponade (balanced salt solution, noble gas, sterile air, or silicone oil) and concurrent phacoemulsification and IOL implantation. Preoperative and postoperative best-corrected Snellen visual acuities were recorded and converted to logMAR equivalents for analysis.

### 2.2. Statistical Analysis

The BCVA was converted to logMAR (logarithm of the minimal angle of resolution) units for statistical analysis. Significant visual improvement, or good visual outcome, was defined as two Snellen lines or more improvement in BCVA (0.3 logMAR units). Changes of BCVA and CFT after surgery were evaluated by paired *t*-test. Groups were compared depending on the characteristics of the variables. The categorical variables were displayed as counts and percentages and analyzed using the *χ*^2^ test or exact Fisher test. The continuous variables were displayed as means ± standard deviation and analyzed using the *t*-test or Mann–Whitney *U* test. The statistical analysis was performed using R software (Version 3.1.1; https://www.R-project.org) or IBM SPSS statistics Version 26. Statistical significance was set at *p* value < 0.05.

## 3. Results

### 3.1. Baseline Characteristics

A total of 36 eyes of 36 patients with high myopia and macular foveoschisis were recruited in this study. The mean age of the subjects was 64.58 ± 6.81 years and the majority were female (86.11%). The mean spherical equivalent refraction was −13.74 ± 4.82 D and the mean duration of symptoms was 21.42 ± 31.83 months. The mean preoperative VA was 1.10 ± 0.61 logMAR units.

Preoperative OCT data indicated comorbidities including FD in 41.67%, lamellar MH in 52.78%, and FTMH in 22.22% of eyes. Discernible VMT was seen in 38.89% and ERM was present in 66.67%. The mean CFT was measured at 427.14 ± 255.91 μm. Most eyes (86.11%) received a gas tamponade. C3F8 was used most often (47.22%), followed by air (36.11%). The remainder received the balanced salt solution or silicone oil (both 8.33%). Combined cataract extraction was performed at the time of vitrectomy in 91.67%, and the others received artificial lens implantation previously. The mean follow-up time was 21.42 ± 31.83 months. Data are shown in Table 1.

The morphological characteristics of the macular were evaluated based on the ATN classification system (Supporting Table 1). The majority groups were A1 and A2 (77.78% and 16.67%, respectively), T2 and T3 (38.89% and 30.56%, respectively), and N0 and N1 (80.56% and 11.11%, respectively). The severe MF group, defined as either A or T components were grade 3 or more, and/or N was grade 2, accounted for 23 (63.89%). Another MTM staging system (MSS), informed by OCT characteristics, prioritizes the assessment of vertical and horizontal retinal tractive forces. In this study, most of the population was characterized by a MSS stage of 3, accounting for 36.12% of the cases, followed by stages 2b (22.22%) and 1b (19.44%). Subsequent ones were represented by 2a (8.33%), 1c (2.78%), and 4c (2.78%), with the least prevalent being stage 4b at 2.78% (Supporting Table 2). In terms of retinal layer status, the outer nuclear layer was influenced in all eyes, becoming the most influenced layer, followed by the inner nuclear layer, retinal nerve fiber layer, and ganglion cell layer, with a proportion of 52.78%, 36.12%, and 19.45%, respectively. The EZ band was disrupted in 18 eyes (50.00%), with 10 (27.78%) and 8 (22.22%) eyes having the disrupted site within or outside the fovea.

## 4. Functional and Anatomical Outcomes

As shown in Table 2, BCVA improved significantly from a preoperative mean of 1.10 ± 0.61 logMAR to a postoperative mean of 0.78 ± 0.58 logMAR (*p*=0.031). The mean CFT decreased from 427.14 ± 255.91 μm preoperatively to 155.85 ± 67.33 μm postoperatively (*p* < 0.001). In total, 29 (80.56%) eyes received improved BCVA after surgery. The good visual group, defined as postoperative BCVA improved by ≥ 2 Snellen lines, occurred in 23 eyes (63.89%). The overall anatomical success rate was 97.22%, with 18 and 16 eyes achieving partial and complete retinal reattachment, respectively (Table 3). One postoperative complication, retinal detachment, occurred in an eye with a FTMH preoperatively. The retina was reattached following a secondary surgical procedure.

## 5. The Short-Term and Long-Term Outcomes

**Figure 1:**
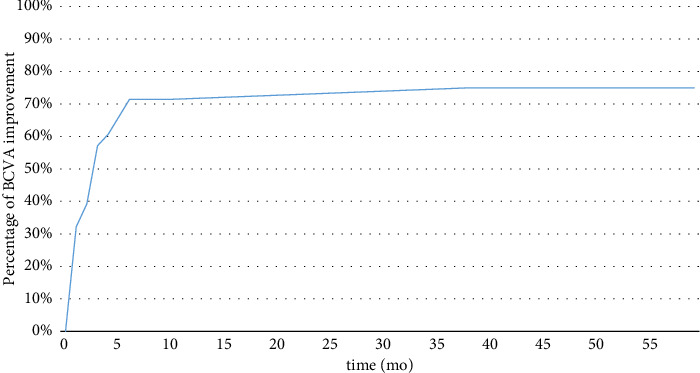
BCVA recovery curve after surgery.

**Figure 2:**
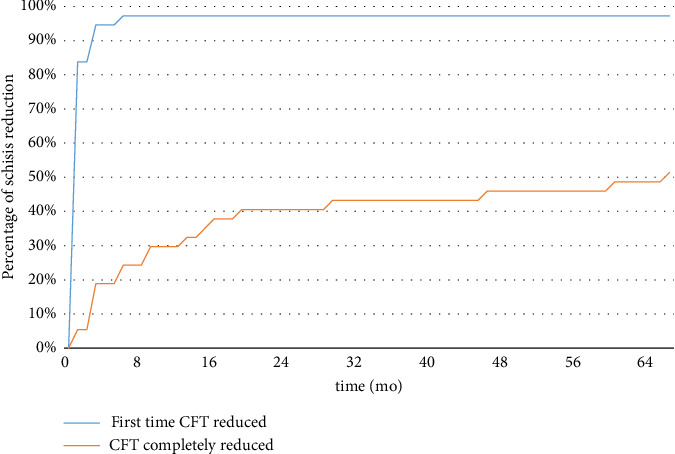
Schisis recovery curve after surgery.

During this period, 20 eyes had a follow-up time of more than 12 months, and 14 eyes had a follow-up time of more than 24 months. Figures 1 and 2 show the recovery curve of the eyes over time post-surgery. Follow-up time was analyzed iteratively, identifying postoperative-12 months as a threshold to influence the resolution of MF significantly, while it did not affect the visual outcome. Patients were divided into two groups, with follow-up time of no more than 12 months (defined as a short-term group, *n* = 16) and more than 12 months (defined as a long-term group, *n* = 20). The BCVA improved one month postoperatively in 98.10% of patients, with no significance between the short-term group and the long-term group (*p* > 0.05, Table 4). In terms of complete retina reattachment, the long-term group showed significantly better anatomical repositioning outcomes compared to the short-term group (*p*=0.005, Table 4).

### 5.1. Prognostic Factors

Univariate variant analysis and lasso regression analyses were performed to identify preoperative prognostic factors for visual and anatomic outcomes following PPV for macular foveoschisis. We analyzed the baseline clinical and OCT characteristics of patients, comparing the good visual group with the bad visual group, and comparing complete retinal reattachment with noncomplete retinal reattachment after the surgery, separately (Table 5).

Smaller height of FD (*p*=0.001), higher IOP (*p*=0.002), and longer follow-up period (*p*=0.002) were associated with better anatomic outcomes, defined as complete reattachment of fovea based on OCT. As postoperative follow-up duration was identified related to anatomical outcomes, adjustment of follow-up time was further performed by eliminating data with follow-up time less than 12 months. Variables were further screened by lasso regression to eliminate variables with no clinical significance, and two factors were incorporated into the mode, the CFT and the minimum diameters of MH. Multiple logistic regression showed only CFT (OR = 1.007, 95% CI = 1.001, 1.013, *p* value = 0.034) remained significant to relate with the complete retina resolution (Table 6).

Results showed that EZ integrity (*p*=0.048) and without history of inner eye surgery except for cataract surgery (*p*=0.015) were significant prognostic factors for good visual outcome (Table 5). Multiple logistic regression showed that disrupted EZ band (OR = 0.239, 95% CI = 0.073, 0.783, *p* value = 0.018) and baseline BCVA (OR = 13.440, 95% CI = 2.185, 82.651, *p* value = 0.005) were significantly related to visual improvement after surgery (Table 7). Stratified analysis for vision outcome was further performed based on EZ integrity. In patients with integrated EZ band, a worse preoperative BCVA (*p*=0.015) resulted to be correlated with an increase of BCVA of more than two Snellen lines. Conversely, in those with disrupted EZ band, all included factors showed no significant difference.

## 6. Discussion

Consistent with previous results [20], this study showed anatomical and functional improvement for MF from vitrectomy with ILM peeling with or without tamponade (*p* < 0.05). Building on previous knowledge, the study tracked the progression of anatomical and functional recovery during the follow-up period after vitrectomy and suggested potential factors that may be associated with surgical outcomes in terms of VA and retinal reattachment.

The study suggested that a trend toward anatomical restoration of retina foveoschisis was observed after 12 months post-operation, and an improvement in VA was noted within the first three months (*p*=0.03, paired *t*-test). The relatively short-term improvement of BCVA is consistent with published reports. Figueroa et al. found VA improved significantly after surgery, and the improvement progressively occurred during the first 6 months of follow-up [13]. However, the postoperative retinal reattachment period was found to be longer than previous reports. For instance, Kim, Lee, and Lee [21], Zheng et al. [22], and Figueroa et al. [13] found macular schisis resolution took around 3–5.1 months on average [23]. One possible explanation for the observed trends is the inclusion of eyes with concomitant MH in our sample. Given the limited sample size, our study did not conduct further statistical analyses to explore differences in MF with or without MH, as such analyses were deemed potentially underpowered. The study's findings indicate that a majority of patients, 98.10%, noted visual improvement one month post-surgery. At the final follow-up, the rate of vision improvement was 83.33%, demonstrating no significant variation between the short-term and long-term groups (*p* > 0.05). In terms of anatomical reattachment, the surgery achieved a success rate of 96.87%, with complete reattachment observed in 45.16% of the eyes. When comparing the long-term and short-term groups, the long-term group tended to have better anatomical outcomes (*p* < 0.05). The study's results indicate the vitrectomy contributing to the improvement of both functional and anatomical outcomes over the long term. This result is consistent with published reports, as reported by Zheng et al. [22], Ho et al. [24], and Zhang et al. [25]. In a study conducted by Ripa et al., the efficacy of MB was investigated in patients at MSS stages 3-4. Upon the final follow-up, 100% of eyes at stages 3b and 4b achieved anatomical resolution, while stage 3a and 4a eyes saw a respective resolution rate of 92.3% and 81.8%. The mean final postoperative BCVA improved by 0.46 logMAR [26]. In this study, the overall BCVA enhancement for eyes at stages 3-4 was averaged at 0.32 logMAR. Additionally, the rate of full retinal recovery in the last follow-up for eyes at stages 3a and 3b was 66.7% each. Due to limitations in sample size, a subgroup analysis for remaining stages could not be performed. It is noteworthy that a direct comparison of the outcomes between the two surgical interventions is not feasible, as the postoperative follow-up durations and baseline VA data vary between studies. Future research necessitates the design of more rigorous, prospective experiments to determine the most suitable surgical approach for eyes at different stages.

The study suggested two factors that might affect visual outcomes: the integrity of the EZ band and the baseline BCVA. It has been reported before that the preoperative VA of patients is positively correlated to their postoperative VA after vitrectomy for MF [11–13, 23]. In contrast to previous studies that examined the linear correlation between pre- and postoperative VA, our research investigated the change in VA for individual eyes before and after surgery. For the purpose of this analysis, eyes were categorized into two groups based on whether the BCVA improved by 2 Snellen lines or more after surgery. The data were then analyzed using univariate and multiple logistic regression analysis. The study's findings suggested that an intact EZ band might be a significant predictive factor for better VA improvement. Li et al. have observed that one of the factors that affect the visual prognosis for the natural course of MTM is the integrity of the EZ band [5]. Our study implied a potential association between the integrity of EZ and postoperative recovery of visual function. The subgroup analysis suggested that, in patients with integrated EZ bands, worse preoperative BCVA might be a predictive factor for better VA improvement. Although several studies have shown a positive linear correlation between BCVA before and after surgery [11, 12, 23, 27], our results suggested that eyes with worse baseline BCVA may have a higher possibility of improving VA by two Snellen lines or more. Fang et al. found significant differences in postoperative BCVA and the rate of primary retinal reattachment between the severe and nonsevere MF groups based on ATN scores, excluding either MH or FD [28]. Similarly, but not identical, our analysis indicated the ATN classification shows a correlation with VA improvement specifically in patients with an intact EZ band.

Furthermore, our analysis suggests that a follow-up period of at least 12 months after surgery may be linked to improved anatomical outcomes. After adjustment of follow-up time, a thicker CFT was identified as a potential predictive factor for complete retinal reattachment after surgery. Dissimilarly, Kim et al. found the presence of FD was associated with poorer anatomical outcomes of PPV in MTM [27]. The difference in results could be due to different VA and FD percentage at baseline. In Kim's research, the baseline BCVA was better, and the presence of FD was six in 40 eyes (15%), while it was 15 in 36 eyes (40.54%) in our study. This variation may contribute to heterogeneity between different study results.

MH is a primary postoperative complication of ILM peeling. In this study, secondary MH was not observed. A secondary MH-associated retinal detachment was noted, and it was successfully resolved through a subsequent surgical intervention that achieved retinal reattachment. A review of literature from 2001 to 2009 reported the incidence of MH as a complication of ILM peeling ranging between 0% and 31.25%, with most studies indicating an incidence rate of 0% [22]. Recent research shows a decrease in the occurrence of postoperative FTMH to 0%–5.1% [23, 29]. In our case series, none of the eyes without FTMH developed secondary MH following surgery. This may be attributed to advancements in surgical techniques, such as gentle membrane peeling and improved peeling skills, including the application of centripetal peeling techniques, all of which contribute to reducing the incidence of postoperative MH. Furthermore, other studies suggested that the technique of sparing the central fovea during ILM dissection effectively reduces the occurrence of postoperative FTMH [30, 31].

There are some inherent limitations in this study. Firstly, the study design was retrospective, not prospective, failing to determine the precise timing of anatomical or functional recovery. While the study provides evidence of significant anatomical recovery on 12 months after vitrectomy, its retrospective nature prevents it from capturing the detailed timeline process. Future studies should conduct prospective design and longitudinal follow-up to more accurately determine the timing of recovery. Secondly, although all participants were operated on by the same experienced surgeon in the same facility, differences in surgical time and postoperative management could not be avoided, which may have caused the results' variabilities. Future studies can further explore the impact of different surgical techniques and postoperative management on the effectiveness of vitrectomy.

In summary, this study presents three major findings. Firstly, PPV with ILM peeling appears to be an effective method for treating highly myopic macular foveoschisis. Secondly, the 12-month postoperative period appears to be a relevant threshold for anatomical restoration. The group with long-term follow-up exhibited improved retinal reattachment outcomes. Lastly, EZ integrity and baseline BCVA are identified as potential prognostic factors for visual improvement. In patients with intact EZ band, poorer preoperative BCVA may be a predictive factor for better visual recovery after the surgery. In contrast, the disrupted EZ band at baseline might predict a poor VA improvement. These findings offer insights that may assist ophthalmic surgeons in considering the decision making and timing of surgery. Additional research is required to develop a precise prediction model that could enhance the outcomes of PPV with ILM peeling therapy for the treatment of macular foveoschisis.

## Figures and Tables

**Table 1:** Baseline characteristics of the patients.

Characteristics	Total cohort (*n* = 36)
Demographic feature	
Age (yr)	64.58 ± 6.81
Male	13.89%
Diopter	−13.74 ± 4.82
Axial length (mm)	29.07 ± 2.02
ACD (mm)	3.50 ± 0.36
Duration of symptoms (m)	21.42 ± 31.83
Preoperative VA (logMAR)	1.10 ± 0.61
Pseudophakic lens	8.33%
Concomitant	
Foveal detachment	41.67%
MH	75.00%
Lamellar MH	52.78%
FTMH	22.22%
Vitreomacular traction	38.89%
ERM	66.67%
OCT feature	
Severe ATN classification	63.89%
Area of MF	7427.85 ± 2355.32
MF more than two layers	27.78%
CFT	427.14 ± 255.91
Disruption of the ellipsoid zone	50.00%
Tamponade	
Silicon oil	8.33%
C3F8	47.22%
Sterile air	36.11%
Balance salt solution	8.33%

**Table 2:** BCVA and CFT of patients before and after surgery for MF.

	Pre-operation	Post-operation (last visit)	*p* value
BCVA (logMAR)	1.10 ± 0.61	0.78 ± 0.58	0.031
CFT (μm)	427.14 ± 255.91	155.85 ± 67.33	0.000

**Table 3:** Functional and anatomical outcomes after surgery.

	BCVA improved (*n*)	BCVA improved more than 2 lines (*n*)
Function	80.56% (29)	63.89% (23)

	**Reduction of schisis (*n*)**	**Resolution of schisis (*n*)**

Anatomy	97.22% (35)	45.71% (16)

**Table 4:** Short and long outcomes after surgery.

	BCVA improved more than 2 lines % *(n*)	Complete retinal reattachment % (*n*)
Short (*n* = 16)	62.50% (10)	25.00% (4)
Long (*n* = 20)	65.00% (13)	70.00% (14)
*p* value	0.881	0.005

**Table 5:** Univariate analysis of factors associated with postoperative visual acuity and retinal reattachment.

	BCVA improved no more than 2 lines (*n* = 14)	BCVA improved more than 2 lines (*n* = 23)	*p* value	Retina not completely reattached (*n* = 20)	Retina completely reattached (*n* = 17)	*p* value
Age (yr)	63.154 ± 6.414	65.391 ± 7.031	0.351	66.100 ± 7.261	62.688 ± 5.873	0.137
Pseudophakic lens	0.231 ± 0.439	≤ 0.001 ± ≤ 0.001	0.015	0.100 ± 0.308	0.062 ± 0.250	0.696
IOP	15.538 ± 2.787	15.761 ± 2.804	0.820	14.450 ± 1.820	17.219 ± 3.005	0.002
Follow-up duration (m)	30.846 ± 26.041	21.652 ± 22.376	0.272	14.700 ± 18.854	37.812 ± 23.597	0.002
Preoperative BCVA (logMAR)	0.871 ± 0.440	1.228 ± 0.654	0.089	1.196 ± 0.676	0.978 ± 0.497	0.288
BCVA at last visit (logMAR)	1.232 ± 0.623	0.473 ± 0.269	≤ 0.001	0.718 ± 0.567	0.844 ± 0.603	0.546
AL (mm)	28.531 ± 2.464	29.356 ± 1.764	0.363	28.723 ± 1.807	29.387 ± 2.224	0.443
ERM (%)	69.2	65.2	0.813	70.0	62.5	0.647
MH (%)	92.3	100.0	0.755	100.0	93.8	0.793
VMT (%)	38.5	39.1	0.970	45.0	31.2	0.415
FD (%)	30.8	47.8	0.333	40.0	43.8	0.827
Severe ATN (%)	53.8	69.6	0.360	60.0	68.8	0.600
Grade of EZ integrity	1.154 ± 0.899	0.565 ± 0.788	0.048	0.950 ± 0.887	0.562 ± 0.814	0.186
Abnormal choroidal surfaces (%)	46.2	30.4	0.590	40.0%	31.2%	0.689
CFT (μm)	399.846 ± 186.872	442.565 ± 290.610	0.637	416.250 ± 305.249	440.750 ± 185.561	0.780
Grade of FD height	1.250 ± 0.500	1.455 ± 0.522	0.510	1.750 ± 0.463	1.000 ± 0.000	0.001

**Table 6:** Multivariate analysis of factors associated with postoperative complete retina resolution.

	OR (95% CI)	*p* value
CFT (μm)	1.007 (1.001, 1.013)	0.034
The minimum diameters of MH (μm)	0.996 (0.992, 1.001)	0.083

**Table 7:** Multivariate analysis of factors associated with postoperative visual acuity.

	OR (95% CI)	*p* value
Pseudophakic lens	0.386 (0.104, 1.435)	0.155
Preoperative BCVA (logMAR)	13.440 (2.185, 82.651)	0.005
Grade of EZ integrity	0.239 (0.073, 0.783)	0.018

## Data Availability

The presented data in this study are available from the corresponding authors upon reasonable request.

## Nomenclature

ACD: Anterior chamber depth
AL: Axial length
BCVA: Best-corrected visual acuity
C3F8: Octafluoropropane
CFT: Central fovea thickness
D: Diopter
ERM: Epiretinal membrane
EZ: Ellipsoid zone
FD: Foveal detachment
FTMH: Full-thickness macular hole
ILM: Internal limiting membrane
IOL: Intraocular lens
IOP: Intraocular pressure
logMAR: Logarithm of the minimal angle of resolution
MB: Macular buckle
MF: Myopic foveoschisis
MH: Macular hole
MSS: MTM staging system
MTM: Myopic traction maculopathy
OCT: Optical coherence tomography
OR: Odds ratio
PPV: Pars plana vitrectomy
VA: Visual acuity
VMT: Vitreomacular traction

## Supporting Information

The supporting tables present baseline classification based on the ATN classification and the MTM staging system of our cohort.
