# Supplementary material for: Factors Affecting the Outcome of Vitrectomy With Internal Limiting Membrane Peeling for Myopic Foveoschisis
Source: J Ophthalmol. 2025 Feb 13;2025:2774963. doi: 10.1155/joph/2774963 (PMC11842140; doi:10.1155/joph/2774963)
Supplement: Supporting Information — Additional supporting information can be found online in the Supporting Information section. [file 2774963.f1.zip › Supplementary table 1.docx]

Supplementary table 1. ATN system classification of the cohort.

| Atrophy (n) | Traction (n) | Neovascularization (n) |
| --- | --- | --- |
| A0 2.78% (1) | T0 0 | N0 80.56% (29) |
| A1 77.78% (28) | T1 11.11% (4) | N1 11.11% (4) |
| A2 16.67% (6) | T2 38.89% (14) | N2a 2.78% (1) |
| A3 0 | T3 30.56% (11) | N2s 5.56% (2) |
| A4 2.78% (1) | T4 11.11% (4) |  |
|  | T5 8.33% (3) |  |

Description:

This table shows the baseline ATN classification of our cohort.
