# Supplementary material for: Factors Affecting the Outcome of Vitrectomy With Internal Limiting Membrane Peeling for Myopic Foveoschisis
Source: J Ophthalmol. 2025 Feb 13;2025:2774963. doi: 10.1155/joph/2774963 (PMC11842140; doi:10.1155/joph/2774963)
Supplement: Supporting Information — Additional supporting information can be found online in the Supporting Information section. [file 2774963.f1.zip › Supplementary table 2.docx]

Supplementary table 2. MTM system classification of the cohort.

| Stage | Percentage of patient (n) |
| --- | --- |
| 1a | 0 (0) |
| 1b | 19.44% (7) |
| 1c | 2.78% (1) |
| 2a | 8.33% (3) |
| 2b | 22.22% (8) |
| 2c | 2.78% (1) |
| 3a | 16.67% (6) |
| 3b | 8.33% (3) |
| 3c | 11.11% (4) |
| 4a | 0 (0) |
| 4b | 2.78% (1) |
| 4c | 5.56% (2) |

Description:

This table shows the baseline classification of the cohort based on the MTM staging system.
